# Engineering a CRISPR Interference System To Repress a Class 1 Integron in Escherichia coli

**DOI:** 10.1128/AAC.01789-19

**Published:** 2020-02-21

**Authors:** Qingyang Li, Peng Zhao, Lili Li, Haifeng Zhao, Lei Shi, Pingfang Tian

**Affiliations:** a School of Food Science and Engineering, South China University of Technology, Guangzhou, China; b College of Life Science and Technology, Beijing University of Chemical Technology, Beijing, China; c Institute of Food Safety and Nutrition, Jinan University, Guangzhou, China

**Keywords:** CRISPR interference, class 1 integron, horizontal gene transfer, antibiotic resistance gene, multidrug resistance

## Abstract

Microbial multidrug resistance (MDR) poses a huge threat to human health. Bacterial acquisition of MDR relies primarily on class 1 integron-involved horizontal gene transfer (HGT) of antibiotic resistance genes (ARGs). To date, no strategies other than the use of antibiotics can efficiently cope with MDR. Here, we report that an engineered CRISPR interference (CRISPRi) system can markedly reduce MDR by blocking a class 1 integron in Escherichia coli. Using CRISPRi to block plasmid R388 class 1 integron, E. coli recombinants showed halted growth upon exposure to relevant antibiotics. A microplate alamarBlue assay showed that both subgenomic RNAs (sgRNAs) R3 and R6 led to 8- and 32-fold decreases in half-maximal inhibitory concentrations (IC_50_) for trimethoprim and sulfamethoxazole, respectively. Reverse transcription and quantitative PCR (RT-qPCR) revealed that the strain employing sgRNA R6 exhibited 97% and 84% decreases in the transcriptional levels of the *dfrB2* cassette and *sul1*, two typical ARGs, respectively. RT-qPCR analysis also demonstrated that the strain recruiting sgRNA R3 showed a 96% decrease in the transcriptional level of *intI1*, and a conjugation assay revealed a 1,000-fold decrease in HGT rates of ARGs. Overall, the sgRNA R3 targeting the 31 bp downstream of the Pc promoter on the *intI1* nontemplate strand outperformed other sgRNAs in reducing integron activity. Furthermore, this CRISPRi system is reversible, genetically stable, and titratable by varying the concentration of the inducer. To our knowledge, this is the first report on exploiting a CRISPRi system to reduce the class 1 integron in E. coli. This study provides valuable insights for future development of CRISPRi-based antimicrobial agents and cellular therapy to suppress MDR.

## Abstract (precis)

Microbial multidrug resistance (MDR) poses a huge threat to human health. Bacterial acquisition of MDR relies primarily on class 1 integron-involved horizontal gene transfer (HGT) of antibiotic resistance genes (ARGs). To date, no strategies other than the use of antibiotics can efficiently cope with MDR. Here, we report that an engineered CRISPR interference (CRISPRi) system can markedly reduce MDR by blocking a class 1 integron in Escherichia coli. Using CRISPRi to block plasmid R388 class 1 integron, E. coli recombinants showed halted growth upon exposure to relevant antibiotics.

## INTRODUCTION

Recent years have witnessed the severe threat of antibiotic-resistant pathogens (ARPs) to human health (1). In the United States, approximately 2 million patients each year are infected with ARPs, resulting in at least 23,000 fatalities, and this situation is getting worse (2). Antibiotics have been extensively harnessed to combat pathogen infections. However, overuse of them accelerates the evolution of microbial multidrug resistance (MDR), and this situation necessitates combinatorial use of antibiotics (1, 3). MDR is largely attributed to horizontal gene transfer (HGT) of antibiotic resistance genes (ARGs), and HGT is typically accomplished by mobile genetic elements (MGEs) through transformation, conjugation, and transduction (3, 4). Among all types of MGEs, mobile integrons (MIs) are commonly found in clinical settings and other circumstances (5–9) and played a crucial role in the early rise of MDR among clinically relevant bacteria in the 1960s (5). Indeed, increasing evidence has shown that the transmission of ARGs among Gram-negative pathogens is frequently brought by the MI-involved HGT of ARGs (9–11).

**FIG 1:**
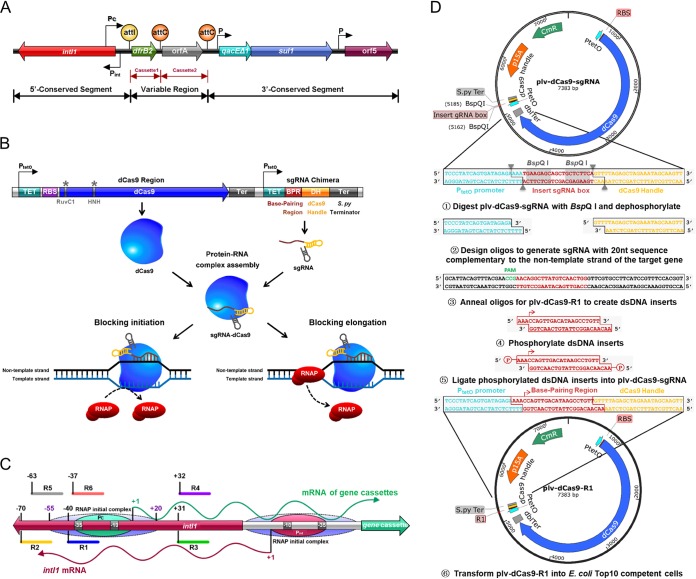
Schematic diagram of engineering a CRISPRi system to repress a class 1 integron in E. coli. (A) Structural diagram of a class 1 integron on conjugative plasmid R388. (B) Harnessing CRISPRi to block transcription initiation and elongation. RNAP, RNA polymerase. (C) Genetic organization of *intI1*, gene cassettes, Pc promoter, P*_int_* promoter, and the binding sites of six sgRNAs. The six sgRNAs were designed for targeting different regions around the Pc promoter, and all target sites were located in the IntI1 integrase-coding region. The transcription start site is labeled as +1. Arabic numerals highlight the distance to the transcription start site of gene cassettes. The dotted purple oval shows the initial RNA polymerase complex. The green oval and red oval represent the Pc promoter of gene cassettes and the P*_int_* promoter of *intI1*, respectively. (D) Protocol for construction of CRISPRi recombinant plasmids. The same strategy is adopted to target the different regions of R388 class 1 integron in E. coli. The nucleotides shown with a gray background are BspQI recognition sequences, and gray triangles indicate the BspQI cutting sites. Red arrowheads indicate the transcription start sites of the base-pairing region of sgRNA.

MIs usually work with transposons, insertion sequences (ISs), and conjugative plasmids (9) and participate in the acquisition, expression, and dissemination of ARGs embedded in gene cassettes (12). Thus, integrons contribute to the transmission of bacterial antibiotic resistance (7). Structurally, almost all integrons are composed of three parts: (i) an *intI* gene (driven by a native promoter P*_int_*), which encodes an IntI integrase belonging to tyrosine recombinase family; (ii) a primary recombination site, *attI*, which serves as both the recognition site of IntI integrase and the receptor site for gene cassettes; and (iii) a Pc promoter within the *intI* coding sequence, which drives the transcription of gene cassettes inserted at the *attI* site (Fig. 1A) (13). The gene cassette usually harbors an open reading frame (ORF) surrounded by an integrase-specific recombination site, *attC* (14, 15). Typically, the integration of the gene cassette into integron is fulfilled by IntI integrase through site-speciﬁc recombination between *attI* and *attC* or between two *attC* sites (16, 17). Once integrated, gene cassettes are expressed under the control of the Pc promoter and transcribed only in a direction opposite to that of *intI* (16, 18). In addition, when subjected to stimuli (e.g., antibiotic selective pressure), the gene cassette located between two adjacent *attC* sites might be excised by IntI integrase, resulting in the rearrangement of internal gene cassettes or gene capture by other integrons (14, 15). The entire process involves integration, expression, and excision of gene cassettes, leading to physiological alternations or even novel genetic traits adapting to environments (3).

Among five classes of MIs (7), class 1 integrons are the most disseminated type in commensals and pathogens of humans and animals (19, 20) and have been found in other ecosystems (21, 22). Structurally, the 3′ conserved segment (CS) in the class 1 integron comprises three elements: *qacE△1* (cationic compound disinfectant resistance gene; GenBank accession number NG_048042), *sul1* (sulfamethoxazole resistance gene; GenBank accession number WP_000259031), and *orf5* (ORF with unknown function) (Fig. 1A) (20). It is extremely challenging to eliminate class 1 integrons as their mobility allows them move onto other recipients to ensure their persistence (8, 13). To date, no efficient approach has been developed to block class 1 integrons. One exception is struvite in combination with a biochar amendment, which has been shown to suppress class 1 integrons in phyllosphere and rhizosphere soils (21). Other exceptions are treatments of residual wastewater solids, such as thermophilic anaerobic digestion, alkaline stabilization, and pasteurization. These approaches can thwart class 1 integrons in wastewater solids-amended soil (22). So far, at least 132 ARG cassettes have been identified which confer resistances to almost all types of antibiotics (14, 19). Therefore, it is highly desirable to come up with an approach suppressing class 1 integrons to counteract the devastating effects of MDRs.

CRISPR technology opens avenues for genome editing and gene regulation (23, 24). In sharp contrast to successful application in fungi, especially Saccharomyces cerevisiae (23, 24), CRISPR editing in bacteria is problematic due mainly to the lack of corresponding DNA repair mechanisms (25). Derived from CRISPR-Cas9, CRISPR interference (CRISPRi) consists mainly of one or several subgenomic RNAs (sgRNAs) and a catalytically dead Cas9 protein (dCas9) lacking endonuclease activity (26). With the goal of knockdown rather than knockout of a gene, CRISPRi technology does not rely on the cellular innate DNA repair machinery and thus is functional in microbes lacking a DNA repair mechanism. The CRISPRi system has been widely harnessed to retard the initiation or elongation of gene transcription (Fig. 1B) (26, 27). Notably, the CRISPRi system can simultaneously upregulate or downregulate multiple genes due to the sgRNAs which lead dCas9 to desired genomic loci (28). Considering that integron-assisted MDR involves a series of genes, CRISPRi may simultaneously interfere with multiple ARGs and thus hold great potential for prevention of bacterial infection and MDR treatment. For instance, simply by encapsulating the dCas9 and a panel of sgRNAs into nanoparticles (29, 30) and incubating these with bacteria for transformation, a wide range of ARGs could be inhibited, achieving desired therapeutic effects.

Given the above information, we conjectured that the CRISPRi system may efficiently reduce MDR by blocking class 1 integrons. Following this assumption, we developed a CRISPRi system in Escherichia coli C600 to curb the class 1 integron on the conjugative plasmid R388 (12). Reverse transcription and quantitative PCR (RT-qPCR) analysis were performed to assess the ability of a CRISPRi system to repress the IntI1 integrase gene and ARGs. A microplate alamarBlue assay (MABA) and growth measurement were performed to dissect the performance of the CRISPRi system in mitigating antibiotic resistance arising from the class 1 integron. Conjugation assays were carried out to disentangle the impacts of the CRISPRi system on HGT of ARGs associated with the IntI1 integrase-mediated integration of ARG cassettes. A titration experiment was performed to determine whether the activity of the CRISPRi system can be controlled by varying the concentration of the inducer. Finally, serial subculture experiments were conducted to investigate the reversibility and hereditary stability of a CRISPRi system. Overall, this study aims to develop a CRISPRi system capable of repressing class 1 integron-aided MDR of E. coli and other bacteria.

## RESULTS

### Reduced antibiotic resistance of E. coli harboring a CRISPRi system.

**FIG 2:**
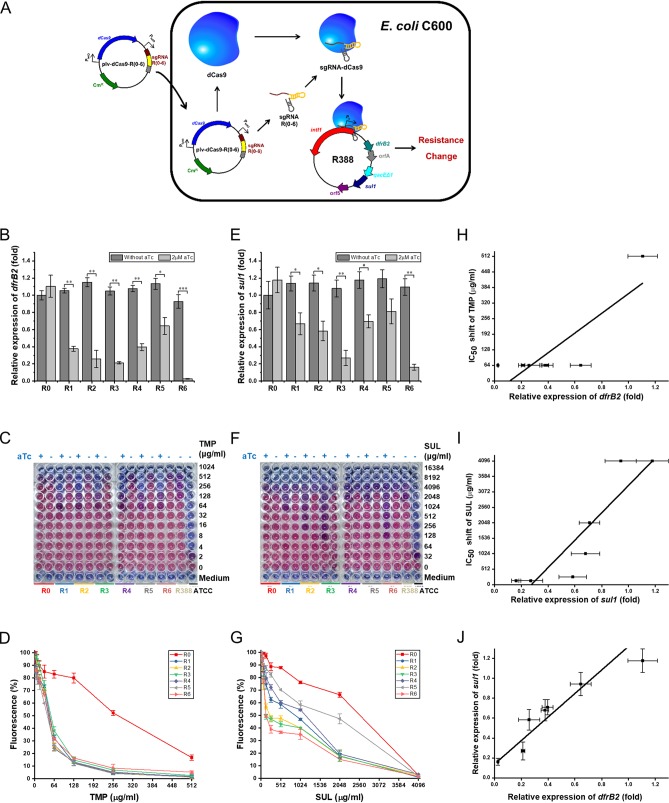
Performance of the CRISPRi system in alleviating the class 1 integron-mediated antibiotic resistance in E. coli. (A) Schematic diagram of a CRISPRi system to attenuate the antibiotic resistance caused by the class 1 integron. (B and E) Transcription of the *dfrB2* cassette (B) or *sul1* (E) in recombinant E. coli strains in the presence or absence of aTc. (C and F) Determination of the IC_50_ of TMP (C) or SUL (F) in recombinant E. coli with and without aTc induction. (D and G) Inhibition of fluorescence in MABA at different concentrations of TMP (D) or SUL (G) with aTc induction. (H) Correlation analysis between the transcription of the *dfrB2* cassette and the IC_50_ of TMP. (I) Correlation analysis between the *sul1* transcription and the IC_50_ of SUL. (J) Correlation analysis of the transcriptional levels of the *dfrB2* cassette and *sul1*. aTc, anhydrotetracycline; IC_50_, half-maximal inhibitory concentration; TMP, trimethoprim; SUL, sulfamethoxazole; MABA, microplate alamarBlue assay; R(0–6), E. coli C600[R388/plv-dCas9-R(0–6)]; R388, E. coli C600(R388); ATCC, E. coli ATCC 25922. All data represent the means ± standard deviations of biological triplicates. *, *P* < 0.05; **, *P* < 0.01; ***, *P* < 0.001.

To inactivate the class 1 integron, we constructed seven recombinant E. coli C600[R388/plv-dCas9-R(0–6)] strains harboring an anhydrotetracycline (aTc)-inducible CRISPRi system driven by the P*_tetO_* promoter (Fig. 2A), whereby R(0–6) stands for different sgRNAs. The strain embracing sgRNA R0 was used as a control. The sgRNAs R1 to R6 were designed to inhibit the R388 class 1 integron, whereby the Pc variant was PcS with an inactive P2 promoter, and the internal ARGs were a *dfrB2* cassette for trimethoprim (TMP) resistance in the variable region and *sul1* for sulfamethoxazole (SUL) resistance in the 3′ CS (Fig. 1A). Sequencing results demonstrated that all CRISPRi recombinant plv-dCas9-R(0–6) plasmids were properly constructed (see Fig. S1 in the supplemental material).

Considering that the CRISPRi system might block the initiation or elongation of gene transcription (Fig. 1B), RT-qPCR was performed to examine the transcriptional levels of the *dfrB2* cassette and *sul1*. Upon aTc induction, the sgRNA R6 targeting the −35 box of the Pc promoter on the *intI1* template strand showed the highest inhibitory efficiency (97% against the *dfrB2* cassette; 84% against *sul1*). In contrast, the sgRNA R5 targeting the upstream region of the Pc promoter on the *intI1* template strand exhibited the lowest inhibition efficacy (37% against the *dfrB2* cassette; 21% against *sul1*). Compared with R6, sgRNAs, including R1, R2, R3, and R4 targeting other regions in the vicinity of the Pc promoter displayed weak inhibition (60 to 80% against the *dfrB2* cassette; 30 to 73% against *sul1*). Collectively, R6 outperformed other sgRNAs in the repression of the *dfrB2* cassette and *sul1*, and the inhibition rates in descending order are R6 > R3 > R2 > R1 > R4 > R5. Furthermore, in the absence of aTc, no significant difference was observed in the mRNA levels of both the *dfrB2* cassette and *sul1* of all CRISPRi strains relative to the levels in the control strain (Fig. 2B and E).

MABA was performed to indicate whether inhibiting the *dfrB2* cassette and *sul1* could lower the phenotypical resistance of E. coli to TMP and SUL, respectively. In the absence of aTc, CRISPRi strains demonstrated no difference in their levels of resistance to TMP and SUL compared with those of the control strain. In contrast, upon aTc induction, the half-maximal inhibitory concentration (IC_50_) of TMP for all CRISPRi strains was decreased by 8-fold (64 μg/ml) relative to that of the control strain (512 μg/ml) (Fig. 2C and D). Unlike the IC_50_ of TMP, the IC_50_s of SUL for all CRISPRi strains were reduced to various degrees. Compared with the IC_50_ of SUL for the control strain harboring sgRNA R0 (4,096 μg/ml), the IC_50_ of SUL for the strain employing R4 or R5 was reduced by 2-fold (2,048 μg/ml), while the IC_50_s for the CRISPRi strains expressing R1 and R2 were reduced by 4-fold (1,024 μg/ml) and 16-fold (256 μg/ml), respectively. Surprisingly, the IC_50_ of SUL for a strain expressing R3 or R6 was decreased by 32-fold (128 μg/ml) (Fig. 2F and G). Clearly, sgRNAs R3 and R6 were more effective than other sgRNAs in mitigating antibiotic resistance, and the order of inhibition rates is R6 ≈ R3 > R2 > R1 > R4 ≈ R5.

Given that the CRISPRi system significantly repressed ARG transcription and antibiotic resistance, we next investigated the correlation between the transcriptional levels of ARGs (*dfrB2* cassette and *sul1*) and the IC_50_s for the corresponding antibiotics (TMP and SUL). Results showed that the transcription of the *dfrB2* cassette was correlated with the IC_50_ of TMP (Pearson correlation coefficient of 0.8436) and that the mRNA level of *sul1* was correlated with the IC_50_ of SUL (Pearson correlation coefficient of 0.9022) (Fig. 2H and I). These results indicated that the reduced resistance to TMP and SUL was largely ascribed to CRISPRi-mediated downregulation of the *dfrB2* cassette and *sul1*, respectively. In addition, there existed a correlation between expression of the *dfrB2* cassette and *sul1* as the Pearson correlation coefficient was up to 0.9414 (Fig. 2J). Hence, we speculate that both the *dfrB2* cassette and *sul1* were controlled by the Pc promoter. Overall, the engineered CRISPRi system significantly reduced antibiotic resistance through blocking the class 1 integron in E. coli.

### Suppressed growth of E. coli harboring a CRISPRi system.

**FIG 3:**
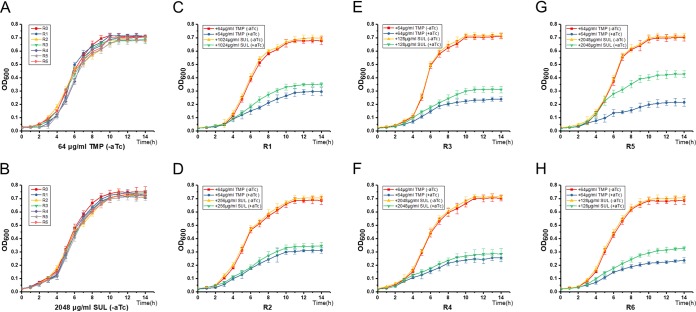
Growth curves of the recombinant E. coli strains harboring the class 1 integron on R388 and with the CRISPRi system. (A and B) The recombinant E. coli strains harboring plasmid R388 and recombinant plasmid plv-dCas9-R(0–6), which were grown in LB medium containing either TMP (A) or SUL (B) without induction of the CRISPRi system. (C to H) The recombinant E. coli strains harboring R388 and plv-dCas9-R(1–6), which were cultivated in LB medium containing TMP/SUL (at the IC_50_) with/without aTc. TMP, trimethoprim; SUL, sulfamethoxazole; IC_50_, half-maximal inhibitory concentration; aTc, anhydrotetracycline; R(0–6), E. coli C600[R388/plv-dCas9-R(0–6)]. All data represent the means ± standard deviations of biological triplicates.

Based on the above-described experimental results, we subsequently investigated the growth of CRISPRi strains in Luria-Bertani (LB) medium containing corresponding antibiotics. We first assessed the effects of an uninduced CRISPRi system on the growth of recombinant E. coli C600[R388/plv-dCas9-R(0–6)] cultivated in LB medium containing TMP/SUL. Not surprisingly, no difference in growth rates was discovered between the CRISPRi strains and the control strain (Fig. 3A and B), indicating that the CRISPRi system has no significant impact on E. coli growth, and leakage expression was substantially avoided through tight control of the P*_tetO_* promoter. Next, we investigated the effects of an aTc-inducible CRISPRi system on the growth of recombinant E. coli C600[R388/plv-dCas9-R(1–6)] incubated in LB medium containing TMP/SUL at the concentration of the IC_50_. Compared to uninduced strains, the aTc-induced CRISPRi strains manifested halted growth from 3 to 14 h (Fig. 3C to H). These results indicated that the CRISPRi system substantially repressed the class 1 integron-aided ARGs and in turn retarded the growth of CRISPRi strains exposed to the antibiotics tested.

### Attenuated HGT of ARGs among E. coli harboring a CRISPRi system.

**FIG 4:**
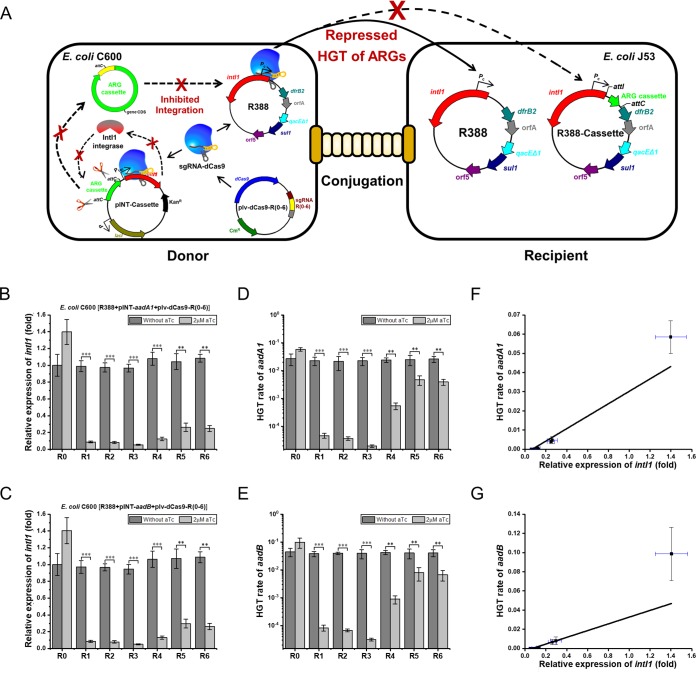
Performance of the CRISPRi system in reducing the class 1 integron-associated HGT of ARGs in E. coli. (A) Schematic diagram of a CRISPRi system to inhibit HGT of ARGs related to the IntI1 integrase-mediated integration of ARG cassettes. (B and C) Transcription of *intI1* in the recombinant E. coli harboring plasmid R388, recombinant plasmid plv-dCas9-R(0–6), and either pINT-*aadA1* (B) or pINT-*aadB* (C). (D and E) HGT rates of *aadA1* (D) or *aadB* (E) associated with the IntI1 integrase-mediated integration of ARG cassettes with/without aTc induction. (F and G) Correlation between *intI1* transcription and the HGT rate of *aadA1* (F) or *aadB* (G). HGT, horizontal gene transfer; ARGs, antibiotic resistance genes; aTc, anhydrotetracycline; R(0–6), E. coli C600[R388/plv-dCas9-R(0–6)/pINT-cassette]. All data represent the means ± standard deviations of biological triplicates. **, *P* < 0.01; ***, *P* < 0.001.

To clarify whether the CRISPRi system impeded the class 1 integron-aided HGT of ARGs, two conjugation models were developed (Fig. 4A). The donor for each model was recombinant E. coli C600[R388/plv-dCas9-R(0–6)] harboring one of the following recombinant plasmids: pINT-*aadA1* for generating an *aadA1* cassette or pINT-*aadB* for generation of an *aadB* cassette. In addition, wild-type E. coli J53 was employed as the recipient in each model. To begin with, we investigated the conjugation between the recipient and the control donor E. coli C600(R388/plv-dCas9-R0/pINT-cassette) without induction of CRISPRi system. The “cassette” indicates different ARG cassettes. The sequencing results of E. coli J53(R388-*aadA1*) and E. coli J53(R388-*aadB*) demonstrated that the two conjugation models allowed for HGT of ARGs among E. coli strains (Fig. S2).

Next, we investigated the HGT rates of *aadA* and *aadB* using the two conjugation models mentioned above. In the absence of aTc, no significant difference was found between the HGT rates of all CRISPRi E. coli C600[R388/plv-dCas9-R(1–6)/pINT-cassette] donor strains and the control donor. Upon aTc induction, all CRISPRi donor strains showed different levels of reduction in HGT rates of ARGs. Compared with the level of the control donor, the CRISPRi donor strain expressing sgRNA R1, R2, or R3 exhibited 1,000-fold inhibition against HGT of both *aadA* and *aadB*, and the CRISPRi donor strain harboring sgRNA R4 led to an approximately 100-fold decrease in HGT. In contrast, the CRISPRi donor strain expressing R5 or R6 displayed only 10-fold decrease in HGT. Strikingly, of all sgRNAs, sgRNA R3 was most efficient in repressing HGT of ARGs, and the inhibitory rates in descending order were R3 > R2 > R1 > R4 > R6 > R5 (Fig. 4D and E).

Since HGT of ARGs in each conjugation model was related to the IntI1 integrase-involved integration of ARG cassettes, the *intI1* transcription in all donors was analyzed by RT-qPCR. The *intI1* transcription of the control donor harboring sgRNA R0 without aTc induction was considered to be 1. Results showed that, in each conjugation model with aTc induction, sgRNAs R1, R2, and R3 targeting the nontemplate strand of *intI1* displayed 90 to 96% inhibition against *intI1* transcription, while the other sgRNAs, R4, R5, and R6, targeting the template strand of *intI1* exhibited only 70 to 85% inhibition against *intI1* transcription. Among all sgRNAs examined, sgRNA R3 was the most effective in the downregulation of *intI1* transcription, and the inhibitory rates in descending order were R3 > R2 > R1 > R4 > R6 > R5. As expected, no significant changes were observed in *intI1* transcription levels when aTc was absent (Fig. 4B and C).

Given that the engineered CRISPRi system significantly attenuated both HGT of ARGs and *intI1* transcription, we next explored the correlation between HGT and *intI1* expression. As shown in Fig. 4F and G, the *intI1* mRNA level was closely correlated with the HGT rate of *aadA1* or *aadB*. The Pearson correlation coefficients were 0.9969 and 0.9944, respectively. In other words, the inhibition efficiency of the CRISPRi system against *intI1* transcription was proportional to the HGT rate of ARGs. Clearly, the CRISPRi system efficiently repressed HGT of ARGs through inhibition of the activity of the class 1 integron in E. coli.

### Titration of CRISPRi system.

**FIG 5:**
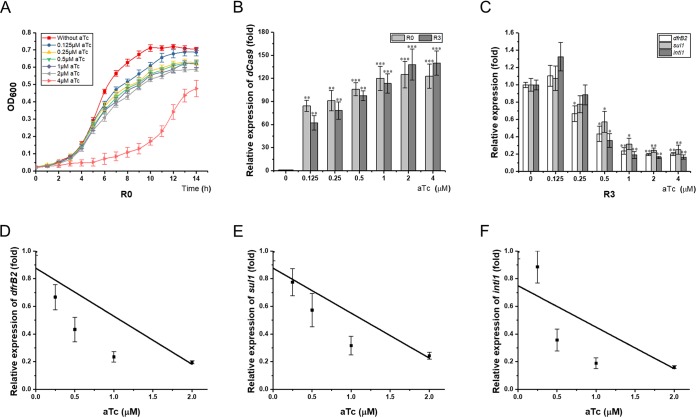
Titration of CRISPRi system in E. coli. (A) Growth curve of the control strain upon induction with 2-fold serial concentrations of aTc ranging from 0 to 4 μM. (B) Transcription of *dcas9* gene in recombinant E. coli harboring plasmid R388 and either recombinant plasmid plv-dCas9-R3 or plv-dCas9-R0 upon induction by aTc ranging from 0 to 4 μM. (C) Transcriptional levels of the *dfrB2* cassette, *sul1*, and *intI1* in the CRISPRi strain harboring sgRNA R3 upon induction by aTc at concentrations ranging from 0 to 4 μM. (D to F) Correlation analysis between the transcription of the *dfrB2* cassette (D), *sul1* (E), or *intI1* (F) and the aTc concentration ranging from 0 to 2 μM. aTc, anhydrotetracycline; R0, E. coli C600(R388/plv-dCas9-R0); R3, E. coli C600(R388/plv-dCas9-R3). Asterisks indicate significant differences between results for aTc-treated and untreated strains (*, *P* < 0.05; **, *P* < 0.01; ***, *P* < 0.001).

To fully assess the engineered CRISPRi system, 2 μM aTc was used to trigger the expression of the *dcas9* gene and sgRNA based on a previously described method (27). To determine the aTc concentration at which the CRISPRi system was adequately triggered while bacterial growth was not negatively affected, we first examined the growth of the control strain E. coli C600(R388/plv-dCas9-R0) cultivated in LB medium containing serial dilutions of aTc. The growth of the control strain was not remarkably halted when the aTc concentration was 0.125, 0.25, 0.5, 1, or 2 μM. In addition, no significant difference in growth rates was observed between aTc-induced and uninduced strains in the first 5 h and after 13 h cultivation (Fig. 5A). However, when 4 μM aTc was used to induce the CRISPRi system, the growth of the control strain was significantly retarded compared to that of the control strain without aTc induction (Fig. 5A). RT-qPCR showed that dCas9 gene transcription relied on aTc induction and that its mRNA level peaked when the aTc concentration reached 2 μM. Interestingly, when 2 μM or 4 μM aTc was used to induce the CRISPRi system, the *dcas9* mRNA level in the CRISPRi strain E. coli C600(R388/plv-dCas9-R3) was slightly higher than that of the control strain (Fig. 5B). Collectively, the appropriate aTc concentration for triggering the CRISPRi system is 2 μM because at this concentration the *dcas9* gene was fully transcribed, and E. coli growth was not significantly halted.

Although the CRISPRi system does not cause double-stranded DNA breaks (DSBs), a low concentration of inducer is preferred as it minimizes the negative influences of the CRISPRi system on the host. To this end, we investigated the influence of serially diluted aTc on the transcriptional levels of the *dcas9* gene, the *dfrB2* cassette, *sul1*, and *intI1* in recombinant E. coli C600(R388/plv-dCas9-R3). We focused on this CRISPRi strain because sgRNA R3 manifested the strongest inhibition on both antibiotic resistance and HGT of ARGs (Fig. 2D to G and Fig. 4D and E). A dose-response correlation was demonstrated between the dCas9 mRNA level and aTc concentration at which the *dfrB2* cassette, *sul1*, and *intI1* were transcriptionally inactivated. While a low concentration of aTc (0.125 or 0.25 μM) did not significantly affect the level of *intI1* transcription compared to that of the control, a high concentration of aTc (0.5, 1, or 2 μM) led to a pronounced increase in the *dcas9* mRNA level, which was correlated with the remarkably repressed transcription of the *dfrB2* cassette, *sul1*, and *intI1*. However, when 4 μM aTc was used, no significant changes were observed compared with levels with 2 μM aTc induction (Fig. 5B and C). Given these results, we investigated the correlation between the transcriptional levels of the *dfrB2* cassette, *sul1*, and *intI1* and aTc at concentrations ranging from 0 to 2 μM. Results demonstrated that the transcription levels of the above three genes were negatively correlated with aTc concentration, with the Pearson correlation coefficients of −0.9243, −0.9199, and −0.8336, respectively (Fig. 5D to F). Taking these results together, CRISPRi efficiency was titratable when the aTc concentration ranged from 0 to 2 μM, and 2 μM aTc led to the strongest CRISPRi efficacy. It is worth noting that moderate, instead of strong, aTc induction is ideal for CRISPRi activity.

### Reversibility and hereditary stability of a CRISPRi system targeting integron.

**FIG 6:**
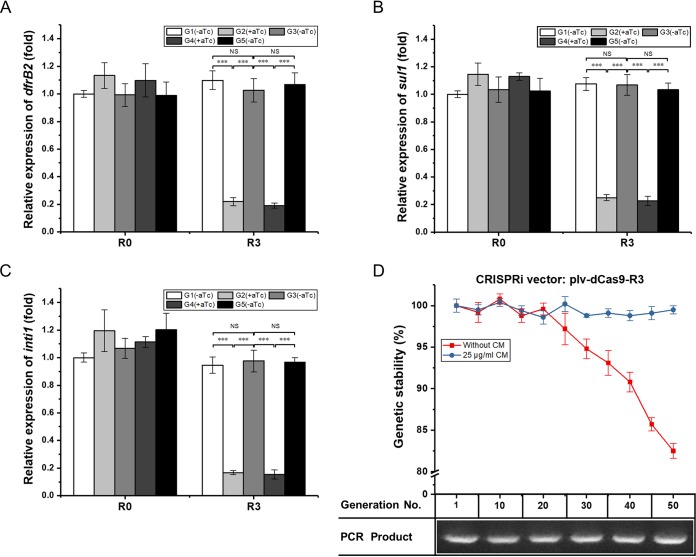
Reversibility and hereditary stability of a CRISPRi system in E. coli. (A to C) Reversibility of transcriptional levels of the *drfB2* cassette (A), *sul1* (B), or *intI1* (C) in different generations of recombinant E. coli harboring a CRISPRi vector exposed to aTc or not. (D) Hereditary stability and PCR analysis of recombinant plasmid plv-dCas9-R3 serially passaged in liquid culture with or without CM. The strain without exposure to CM was used as the control. aTc, anhydrotetracycline; CM, chloramphenicol; R0, E. coli C600(R388/plv-dCas9-R0); R3, E. coli C600(R388/plv-dCas9-R3). NS, not significant (*P* > 0.05); ***, *P* < 0.001.

To determine whether the CRISPRi system is reversible in E. coli, the strain E. coli C600(R388/plv-dCas9-R3) and the control strain E. coli C600(R388/plv-dCas9-R0) were incubated in aTc-lacking LB medium until mid-exponential phase, and the resultant strains were considered the first generation. Subsequent generations were acquired as described in Materials and Methods. The first generation of E. coli C600(R388/plv-dCas9-R3) was induced by aTc, resulting in the second generation. RT-qPCR analysis showed that the second-generation strain demonstrated a dramatic decrease in the transcriptional levels of the *dfrB2* cassette, *sul1*, and *intI1* compared with those of the first-generation strain. Notably, when aTc was removed to acquire the third generation, the transcriptional levels of the *dfrB2* cassette, *sul1*, and *intI1* were restored to those of the first-generation strain. The same manipulation was repeated for the fourth and fifth generations (Fig. 6A to C). For each generation of the control strain, whether aTc was present or absent, no significant change was observed in the mRNA levels of the *dfrB2* cassette, *sul1*, and *intI1* (Fig. 6A to C). The above results indicated that the activity of the CRISPRi system was controllable and reversible in E. coli.

Apart from reversibility, we also investigated the hereditary stability of the CRISPRi system. To do so, we focused on the hereditary stability of recombinant E. coli C600(R388/plv-dCas9-R3), which was continuously cultured in LB liquid medium containing chloramphenicol (CM), as appropriate. The same strain continuously cultured in LB liquid medium without CM was employed as the control. After 50 generations of passage in LB liquid medium containing CM, we found that all cells harbored recombinant plasmid plv-dCas9-R3. In contrast, in the absence of CM, only 82.5% of cells harbored plv-dCas9-R3 (Fig. 6D and Fig. S3). PCR analysis showed that the amplified DNA band was our desired 522-bp fragment (Fig. 6D), confirming that the colony on the LB-CM plate included plv-dCas9-R3. The above results indicated that the plv-dCas9-R3 plasmid in CM-lacking LB medium was less stable than that in CM-containing LB medium. In other words, CM is crucial for the maintenance of the engineered CRISPRi system.

## DISCUSSION

In this work, a CRISPRi system was developed to repress the mobile class 1 integron on conjugative plasmid R388 in E. coli (Fig. 1). The transcriptional levels of the *dfrB2* cassette and *sul1* were downregulated by 97% and 84%, respectively (Fig. 2B and E). As a result, the IC_50_ values for TMP and SUL were reduced by 8- and 32-fold, respectively (Fig. 2C, D, F, and G). Meanwhile, the strains showed reduced growth relative to that of the control strain harboring a CRISPRi vector but without a targeting sequence (Fig. 3C to H). Notably, the mRNA level of *intI1* was downregulated by 96% (Fig. 4B and C), and the HGT rates for both *aadA1* and *aadB* were reduced by 1,000-fold (Fig. 4D and E). Among all sgRNAs examined, sgRNA R3 was most effective in reducing the activity of the class 1 integron. Furthermore, the engineered CRISPRi system is reversible (Fig. 6A to C) and genetically stable (Fig. 6D and Fig. S3), and its activity is titratable by varying the aTc concentration (Fig. 5). Based on a comprehensive consideration of the above results, we recognized that the 31 bp downstream of the Pc promoter on nontemplate strand of *intI1* is the ideal target of the CRISPRi system. To our knowledge, this is the first report of harnessing a CRISPRi system to thwart the class 1 integron in E. coli.

In an effort to efficiently block the class 1 integron, we designed and chemically synthesized a total of six sgRNAs. We found that all six sgRNAs (R1 to R6) significantly reduced the IC_50_ of TMP, indicating that Pc promoter and its vicinity are appropriate targets of the CRISPRi system (Fig. 2C and D). Of six sgRNAs tailored to block the integron, the sgRNA R3 targeting the 31 bp downstream of the Pc promoter on the nontemplate strand of *intI1* displayed the strongest inhibition on the class 1 integron. Although sgRNA R6 showed strongest inhibition on both the *dfrB2* cassette and *sul1* (Fig. 2B and E), sgRNA R3 not only displayed the strongest inhibition against HGT of ARGs but also resulted in the lowest IC_50_ values (similar to those of sgRNA R6) for both TMP and SUL (Fig. 2C, D, F, and G). Upon comprehensive consideration, we conclude that sgRNA R3 is more effective than sgRNA R6 in mitigating the integron.

Apart from the screening of powerful sgRNAs, we tried to disentangle the responses of different regions of the class 1 integron to the CRISPRi system. We found that sgRNAs R1, R2, and R3 targeting the nontemplate strand of *intI1* displayed higher inhibition against HGT of ARGs than sgRNAs R4, R5, and R6 targeting the template strand of *intI1* (Fig. 4D and E). This result is consistent with previous study (26). For the CRISPRi strain recruiting sgRNA R3, the correlation coefficient between aTc concentration and *intI1* transcription was less than that between aTc concentration and the *dfrB2* cassette transcription (Fig. 5D to F). This indicated that the promoter of the *dfrB2* cassette is more easily inhibited than the coding sequence of *intI1*. With regard to ARGs in the class 1 integron, the *dfrB2* cassette was transcriptionally synchronized with *sul1* (Fig. 2J), suggesting that the Pc promoter drove the transcription of both the *dfrB2* cassette and the *sul1* gene located in the 3′ CS of the integron. This finding is consistent with the notion that *sul1* is originally an inserted ARG (12), and it may permanently reside in the integron if it benefits the survival of the host cell (31). Unlike the IC_50_ of TMP, that of SUL for all CRISPRi strains was reduced in various degrees (Fig. 2C and F). A previous study (6) suggests that the two *attC* sites between the Pc promoter and *sul1* (Fig. 1A) might form a stable stem-loop which impedes ribosome progression along polycistronic RNA and thus reduces *sul1* translation.

This work provides insights for future management of MDR pathogens. For instance, based on experimentally validated sgRNA R3, a vehicle encompassing sgRNA R3 and the dCas9 could be prepared and delivered to desired settings and subsequently respond to cues such as pH (30), light (29, 32), or magnetism (33) for eliciting a CRISPRi system. In fact, CRISPR delivery systems have been under construction recently (29, 30, 32, 33). For instance, one group from Nanjing University attempted to cure a tumor by a near-infrared (NIR) light-responsive nano carrier of CRISPR-Cas9 (32). Shortly afterwards, another group from Rice University reported spatial control of CRISPR editing through an artificial magnetic-field-driven baculoviral vector (33). These interdisciplinary studies are broadening the applications of the CRISPR system (34). In addition to the aforementioned nanoparticles (32, 33), the CRISPR system can be delivered by conjugative plasmids (35, 36), phagemids (35), mobile genomic islands (37), or transposons (38, 39). Given this information, we envision that the CRISPR system may be fused to an integron, resulting in a chimeric integron which is mobile. To do so, the original 3′ CS genes of integrons can be replaced by Cas9/dCas9 genes and sgRNAs that specially target virulence and biofilm-associated genes. More broadly, this artificial integron could be harnessed for microbial breeding (40), targeted killing of pathogens (41), epidemiological forecasting when combined with fluorescent label (42), and monitoring of anthropogenic pollution (43). Overall, this study provides valuable insights for both mitigation and utilization of a class 1 integron.

## MATERIALS AND METHODS

### Strains, vectors, and medium.

The strains and vectors used in this study are listed in Table S1 in the supplemental material. Wild-type E. coli C600 was used as the host strain for harboring vectors, and E. coli J53 was employed as the recipient strain to examine bacterial conjugation. Conjugative plasmid R388 (GenBank accession number NC_028464) harbors a class 1 integron. Vector plv-dCas9-sgRNA (27) was used as the backbone of CRISPRi system. The engineered plasmid pINT-cassette was used for gene cassette insertion and IntI1 integrase expression. All E. coli strains were grown in Luria-Bertani (LB) medium containing 10 g/liter tryptone, 5 g/liter yeast extract, 10 g/liter sodium chloride, and antibiotics and inducers as appropriate. Shake-flask cultivation was carried out in a rotatory shaker at 180 rpm. Positive clones were screened by LB plates (LB medium with 1.5% agar) containing antibiotics and inducers, as appropriate, at 37°C.

### Development of a CRISPRi system to inhibit a class 1 integron in E. coli.

Wild-type E. coli C600 was transformed with conjugative plasmid R388, resulting in recombinant E. coli C600(R388). To inhibit R388 class 1 integron, E. coli C600(R388) was transformed with the CRISPRi vector plv-dCas9-R(0–6), resulting in recombinant E. coli C600[R388/plv-dCas9-R(0–6)], where the plv-dCas9-R(0–6) plasmids were derived from plv-dCas9-sgRNA (27) and where R(0–6) stands for different sgRNAs. The plasmid plv-dCas9-sgRNA (27) contains an inactive dCas9 gene from Streptococcus pyogenes and an sgRNA chimera, both of which are driven by an aTc-inducible P*_tetO_* promoter. The sgRNA chimera consists of three parts: (i) a 20-bp DNA fragment complementary to the target locus, named the base-pairing region (BPR); (ii) a 42-bp hairpin region, named dCas9 handle (DH), for dCas9 binding; and (iii) a 40-bp terminator designated *rrnB* (Ter) (Fig. 1B) (26). The BspQI sites in plasmid plv-dCas9-sgRNA were used for directional cloning of any sgRNA into this vector without leaving a scar. After plv-dCas9-sgRNA was digested by BspQI, two different 3-nucleotide (nt) overhangs were formed, and the BspQI sites were removed from the vector. Taking the construction of recombinant plasmid plv-dCas9-R1 as an example, two complementary oligonucleotides, 20 bases of sequence homologous to the target loci and 3 bases at the 5′ end of each oligonucleotide matching the BspQI-digested vector, were annealed, phosphorylated, and cloned into plv-dCas9-sgRNA (Fig. 1D). This protocol allows for plug-and-play of sgRNAs.

The target locus should be immediately downstream of the sequence CCX, the reverse complement of an NGG protospacer adjacent motif (PAM) sequence (44). According to this, we selected the CCX around the Pc promoter to determine the target sequence in R388 class 1 integron. A total of seven sgRNAs were designed. Both sgRNA R1 and R6 were designed to target the −35 box of the Pc promoter, the sgRNAs R2 and R5 target the upstream region of the Pc promoter, and sgRNA R3 and sgRNA R4 target the downstream region of the Pc promoter as well as the region between the transcription start site and the initiation codon of the *dfrB2* cassette. Notably, all of the above sgRNAs were designed to target the coding region of the IntI1 integrase. While the sgRNAs R1, R2, and R3 were designed to target the nontemplate strand of *intI1*, sgRNAs R4, R5, and R6 were designed to target the template strand of *intI1* (Fig. 1C). The sgRNA R0 without targeting any sequence in all experimental strains was prepared as the control. In addition, a BLAST search was performed to examine the specificity of sgRNAs. Subsequently, all oligonucleotides listed in Table S2 for generating sgRNAs were synthesized to form recombinant plv-dCas9-R(0–6) plasmids, which were individually transformed into competent E. coli cells and confirmed by colony PCR and sequencing.

### Construction of recombinant plasmids for gene cassette insertion and IntI1 integrase expression.

The recombinant plasmid pINT-cassette harbors a promoter-lacking ARG cassette and an *intI1* gene (encoding class 1 integron integrase IntI1; GenBank accession number WP_000845048) with an isopropyl-β-D-thiogalactopyranoside (IPTG)-inducible *tac* promoter. Notably, the ARG embedded in the gene cassette follows the same coding orientation as *intI1*. The *tac* promoter along with multiple cloning sites were synthesized and inserted into vector pET-28a between BglII and NocI sites to generate vector pEtac-28a. In this process, one BmgBI site was introduced into pEtac-28a to facilitate subsequent insertion of an ARG cassette (Fig. S4A). The *intI1* was amplified by PCR from plasmid R388 with primers NdeI-intI1-F/intI1-XhoI-R and then cloned into NdeI-XhoI doubly digested vector pEtac-28a, resulting in vector ptacINT (Fig. S4B). Next, the *aadA1* cassette was amplified from the *Salmonella* sp. strain s010 class 1 integron (GenBank accession number AB285480) with primers BglII-aadA1-F/aadA1-BmgBI-R and then inserted into BglII-BmgBI doubly digested vector ptacINT, resulting in recombinant plasmid pINT-*aadA1* (Fig. S4C). The *aadB* cassette from the *Salmonella* sp. strain s084 class 1 integron (GenBank accession number AB285479) was amplified by PCR with BglII-aadB-F/aadB-BmgBI-R and then cloned into BglII-BmgBI doubly digested vector ptacINT, yielding recombinant plasmid pINT-*aadB* (Fig. S4D). This framework allowed for the generation of an episomal ARG cassette mediated by IntI1 integrase following IPTG induction. All recombinant plasmids were transformed into competent cells and confirmed by colony PCR and sequencing. All primers for pINT-cassette construction are listed in Table S2.

### Determination of antibiotic susceptibility.

The recombinant E. coli C600[R388/plv-dCas9-R(0–6)] strains were examined for their antibiotic susceptibility by MABA (45). The strain E. coli ATCC 25922 was employed as the quality control. Twofold serial dilutions of specific antibiotics were carried out in a 96-well plate containing 100 μl of Muller-Hinton (MH) medium per well. The aforementioned strains were first precultured at 37°C with 180 rpm shaking until an optical density at 600 nm (OD_600_) of 0.6 prior to inoculation in MH medium. The turbidity was adjusted to McFarland standard no. 0.5 and further diluted 100-fold. Next, 100 μl of broth was added to each well. In addition, the growth control of each strain and an aseptic control were employed. To trigger the CRISPRi system, aTc was added to a final concentration of 2 μM. Each strain grown in medium devoid of aTc was used as a reference. Plates were cultured at 37°C for 12 h, at which point 20 μl of alamarBlue (Invitrogen) was added to each well. Plates were incubated at 37°C for an additional 6 h and then analyzed with a fluorescence plate reader (excitation at 540 nm/emission at 595 nm; Elx800 plate reader). The lowest concentration of an antibiotic that reduced the fluorescence level to 50% of that of the growth control devoid of antibiotics was considered the IC_50_. The IC_50_ experiments were performed in triplicate.

### RNA Isolation and RT-qPCR.

Tested strains were grown in LB medium for 8 h and then harvested for subsequent RNA extraction using RNAiso Plus (TaKaRa). Absorbance values at 260 and 280 nm were measured by a Nanodrop instrument to determine the quantity and purity of RNA. Quantitative PCR (qPCR) of RNA without reverse transcription (RT) was performed to exclude the effects of genomic DNA contamination. RT-qPCR was carried out using a PrimeScript RT reagent kit (TaKaRa) and SYBR Premix Ex Taq II (TaKaRa). The qPCR was conducted using an Applied Biosystems 7300 real-time PCR system with Relative Expression Software Tool 2009, version 2.0.13. The RT reaction was performed with 100 ng of RNA isolated as described above in a 20-μl final reaction volume. The primers for RT-qPCR analysis were designed by Primer Premier, version 5.0, software to generate amplicons of 90 to 110 nt (Table S2). The slope of the standard curve of serially diluted cDNA showed that the amplification efficiencies of all primer pairs were higher than 99%. The qPCR data were analyzed using the ΔΔ*C_T_* method (where *C_T_* is threshold cycle) with E. coli 16S rRNA as an internal control. All samples were prepared in triplicate to investigate each target sequence.

### Determination of bacterial growth.

The recombinant E. coli C600[R388/plv-dCas9-R(0–6)] was first precultured overnight in LB medium containing 25 μg/ml CM and 64 μg/ml TMP (at 37°C with shaking at 180 rpm) and then diluted to an OD_600_ of 0.01. To elucidate the effects of a CRISPRi system on cell growth, 200 μl of broth was added into a 96-well plate containing 64 μg/ml TMP and 2,048 μg/ml SUL, respectively, but without aTc. To disentangle the effects of the CRISPRi system on the growth of recombinant E. coli C600[R388/plv-dCas9-R(1–6)], 200 μl of broth was inoculated into a 96-well plate containing 2 μM aTc and TMP or SUL at the IC_50_ level. Each strain grown in medium devoid of aTc was used as a control. To investigate the growth of the control strain E. coli C600(R388/plv-dCas9-R0) subjected to different degrees of CRISPRi induction, 200 μl of broth was cultivated in a 96-well plate containing 64 μg/ml TMP and 2-fold serial dilutions of aTc from 0.125 to 4 μM. All 96-well plates were placed in a shaker at 37°C and cultivated for 14 h. Cell concentration was measured every 1 h using a microplate reader (Elx800).

### Bacterial conjugation.

E. coli C600[R388/plv-dCas9-R(0–6)] was transformed with the aforementioned recombinant plasmid pINT-cassette, yielding donor E. coli C600[R388/pINT-cassette/plv-dCas9-R(0–6)]. Since plasmids R388, plv-dCas9-R(0–6), and pINT-cassette possess distinct replication origins, they are compatible with each other in E. coli. To examine the HGT of ARGs from the above donor strain to the recipient E. coli J53, conjugation experiments (17) were performed by the method of filter mating as described previously (35). The donors were incubated overnight in LB broth at 37°C and then diluted 100-fold with fresh medium containing 25 μg/ml CM, 25 μg/ml kanamycin (KAN), and 32 μg/ml TMP. After 4 h of cultivation, 2 μM aTc and 0.5 mM IPTG were simultaneously added into the donor to facilitate the integration of ARG cassettes into the R388 class 1 integron upon CRISPRi induction. Each donor received only 0.5 mM IPTG for use as a reference. The recipient was cultivated overnight in LB medium and then diluted 100-fold with fresh medium containing 100 μg/ml sodium azide (NaN_3_). When the OD_600_ reached 1.0, the donor and recipient strains were pelleted and resuspended with sterile phosphate-buffered saline (PBS). Subsequently, the donor and recipient strains were mixed at a ratio of 3:1. After the mixture was pelleted and resuspended in 20 μl of PBS, filter mating was carried out by spotting the mixture onto a 0.45-μm-pore-size filter (Millipore) on an LB plate. After 16 to 18 h of conjugation, cells were harvested by vigorously vortexing the filter in 1 ml of aseptic PBS. The mixture was serially diluted using PBS and plated onto LB agar containing 1,024 μg/ml SUL and 100 μg/ml NaN_3_ for screening total transconjugants with R388 plus those with R388-*aadA1* or R388-*aadB*. As the genes *aadA1* and *aadB* confer resistance to streptomycin (STR) and gentamicin (GEN), respectively, the mixture was transferred to an LB plate containing 1,024 μg/ml SUL, 25 μg/ml STR, and 100 μg/ml NaN_3_ to screen for transconjugant E. coli J53(R388-*aadA1*). In parallel, the mixture was plated onto an LB plate containing 1,024 μg/ml SUL, 25 μg/ml GEN, and 100 μg/ml NaN_3_ to screen for transconjugant E. coli J53(R388-*aadB*). The HGT rate of *aadA1* or *aadB* was defined as the ratio of transconjugants with R388-*aadA1* or R388-*aadB* to total transconjugants. The donor and recipient strains were independently screened on triple-resistance plates to exclude the effects of spontaneous mutation on the mating assay. To unravel whether ARGs could move from the donor to the recipient, a conjugation experiment was first performed between the recipient E. coli J53 and the control donor E. coli C600(R388/plv-dCas9-R0/pINT-cassette) without CRISPRi induction. The genetic identity of transconjugants with R388-*aadA1* or R388-*aadB* was investigated by PCR using the specific primers Integration-CX-F/aadA1-CX-R to amplify *intI1-attI-aadA1* in transconjugant E. coli J53(R388-*aadA1*) and Integration-CX-F/aadB-CX-R to amplify *intI1-attI-aadB* in transconjugant E. coli J53(R388-*aadB*). All primers used for sequencing are listed in Table S2.

### Serial passage experiments.

To determine whether this CRISPRi system is reversible, the first generation of recombinant E. coli C600(R388) harboring either recombinant plasmid plv-dCas9-R0 or plv-dCas9-R3 was cultivated in LB medium without aTc until the OD_600_ reached 1.0. Next, 1% broth was transferred to LB medium containing 2 μM aTc, yielding the second generation of E. coli strains, and incubated until an OD_600_ of 1 was reached, and the cells were washed with fresh PBS buffer. Next, 1% of a second-generation strain was grown in LB medium devoid of aTc to acquire the third generation. After the culture reached an OD_600_ of 1.0, the third-generation strain was pelleted, resuspended in aseptic PBS, and diluted 100-fold with aTc-containing LB medium to acquire the fourth generation, which was incubated as described for the previous rounds to form the fifth generation. After 8 h of cultivation, the transcriptional levels of the *dfrB2* cassette, *sul1*, and *intI1* in different generations of strains were tested.

To investigate the hereditary stability of the CRISPRi system, the recombinant plasmid plv-dCas9-R3 was tested as reported previously (46), with minor modifications. Plasmid stability was measured by subculture in liquid medium. The strain E. coli C600(R388/plv-dCas9-R3) was precultured overnight in LB medium containing 25 μg/ml CM (37°C and shaking at 180 rpm) and subsequently diluted 100-fold with fresh medium containing 25 μg/ml CM. From that, 1 ml was diluted in CM-lacking LB medium and was used as the reference. Serial passaging was performed every 12 h as follows. The CM-treated strain was sampled and diluted 100-fold by fresh medium containing CM. In contrast, the reference was sampled and diluted by LB medium without CM. Subsequently, aliquots of samples were obtained from the 1st, 10th, 20th, 30th, 40^th^, and 50th generations and serially diluted, spread, and enumerated in LB plates containing 25 μg/ml CM. The LB plates without CM were used as controls. The hereditary stability of vector plv-dCas9-R3 was plotted as a fraction of plasmid-containing cells for each 10 generations, which was evaluated by the colony number on LB-CM plates divided by that on LB plates without CM. The single colony surviving on LB-CM plates of different generations was confirmed by PCR using primers BspQI-R3-F/sgRNA-CX-R. Absence of a PCR product was considered plasmid loss. The experiments investigating reversibility and hereditary stability were performed in triplicate.

### Statistical analysis.

An *F* test of two samples for variance was performed. Significance of the differences (*P* values) was calculated using unpaired two-tailed *t* tests for equal or unequal variance. All tests were performed by the data analysis software GraphPad Prism, version 5.0.

## Supplementary Material

Supplemental file 1
