# Supplementary material for: Engineering a CRISPR Interference System To Repress a Class 1 Integron in Escherichia coli
Source: Antimicrob Agents Chemother. 2020 Feb 21;64(3):e01789-19. doi: 10.1128/AAC.01789-19 (PMC7038292; doi:10.1128/AAC.01789-19)
Supplement: Supplemental file 1 [file AAC.01789-19-s0001.pdf]

Supplemental Material

Engineering CRISPR Interference System to Repress Class 1 Integron in *E. coli*

Qingyang Li[a], Peng Zhao[b], Lili Li[c], Haifeng Zhao[a], Lei Shi[c*], Pingfang Tian[b*]

[a] School of Food Sciences and Technology, South China University of Technology, Guangzhou 510641, China

[b] College of Life Science and Technology, Beijing University of Chemical Technology, Beijing 100029, China

[c] Institute of Food Safety and Nutrition, Jinan University, Guangzhou 510632, China

[*]Corresponding author

E-mail: leishi@jnu.edu.cn (LS)

E-mail: tianpf@mail.buct.edu.cn (PT)

Legends

Fig. S1 Partial sequencing results of seven CRISPRi recombinant plasmids. The base-pairing regions of all CRISPRi recombinant plasmids plv-dCas9-R(0-6) were the same as designed, whereby "R(0-6)" represent different sgRNAs.

Fig. S2 Partial sequencing results of ARG cassettes integrated into R388 class 1 integron. (**A-B**) Partial sequence of integrated fragment *intI1-attI-aadA1* (A) or *intI1-attI-aadB* (B) through conjugation from the control donor *E. coli* C600(R388+plv-dCas9-R0+pINT-cassette) to the recipient *E. coli* J53 without CRISPRi induction.

Fig. S3 Plasmid loss of the CRISPRi recombinant plasmid plv-dCas9-R3 in serial passage. (**A**) Colony counts were enumerated on LB+CM plates to assess plasmid loss of every ten generations continuously passaged in liquid medium containing CM. (**B**) Colony counts were enumerated on LB+CM plates to determine plasmid loss of every ten generations in serial passage without exposure to CM. CM, chloramphenicol; G, generation.

Fig. S4 Schematic outline of construction of pINT-cassette. (**A**) Sequence of BmgBI-P_{tac} for recombinant plasmid pINT-cassette. (**B**) Schematic diagram of pINT-cassette. (**C-D**) The resulting recombinant plasmids pINT-*aadA1* (C) and pINT-*aadB* (D).

Table S1 Strains and vectors used in this study.

Table S2 Oligonucleotides and primers used in this study.

Table S3 IC_{50} of TMP and SUL in recombinant *E. coli* with/without aTc induction.

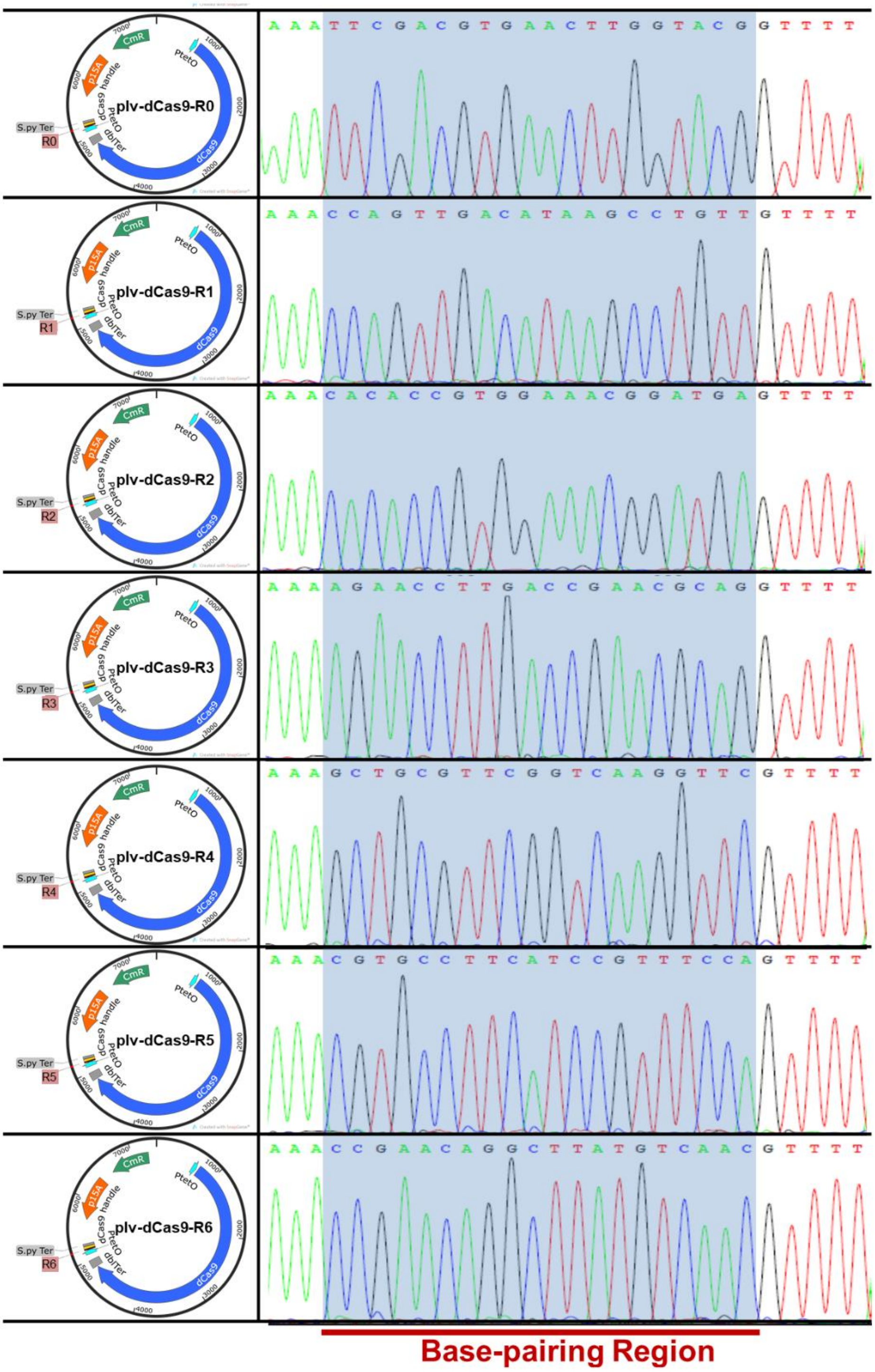

Fig S1 Partial sequencing results of CRISPRi recombinant plasmids. The base-pairing regions of plv-dCas9-R(0-6) were the same as designed, whereby "R(0-6)" represent different sgRNAs.

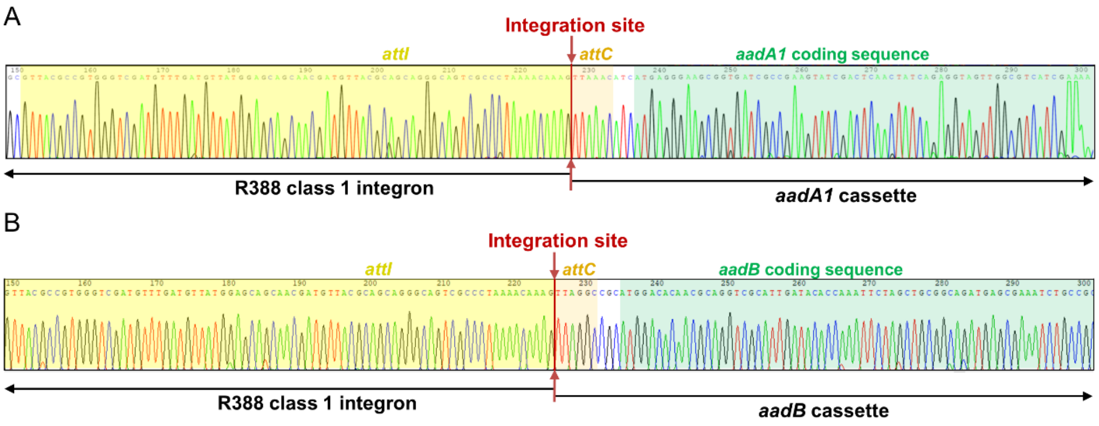

Fig S2 Partial sequencing results of ARG cassettes integrated into R388 class 1 integron. (**A-B**) Partial sequence of integrated fragment *intI1-attI-aadA1* (A) or *intI1-attI-aadB* (B) through conjugation from the control donor *E. coli* C600(R388+plv-dCas9-R0+pINT-cassette) to the recipient *E. coli* J53 without CRISPRi induction.

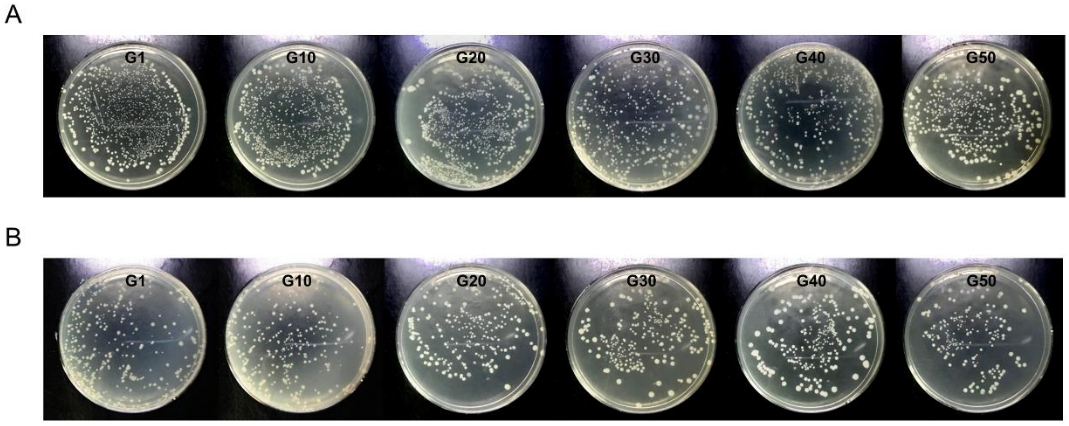

Fig S3 Plasmid loss of CRISPRi recombinant plasmid plv-dCas9-R3 in serial passage. (**A**) Colony counts were enumerated on LB+CM plates to assess plasmid loss of every ten generations continuously passaged in liquid medium containing CM. (**B**) Colony counts were enumerated on LB+CM plates to determine plasmid loss of every ten generations in serial passage without exposure to CM. CM, chloramphenicol; G, generation.

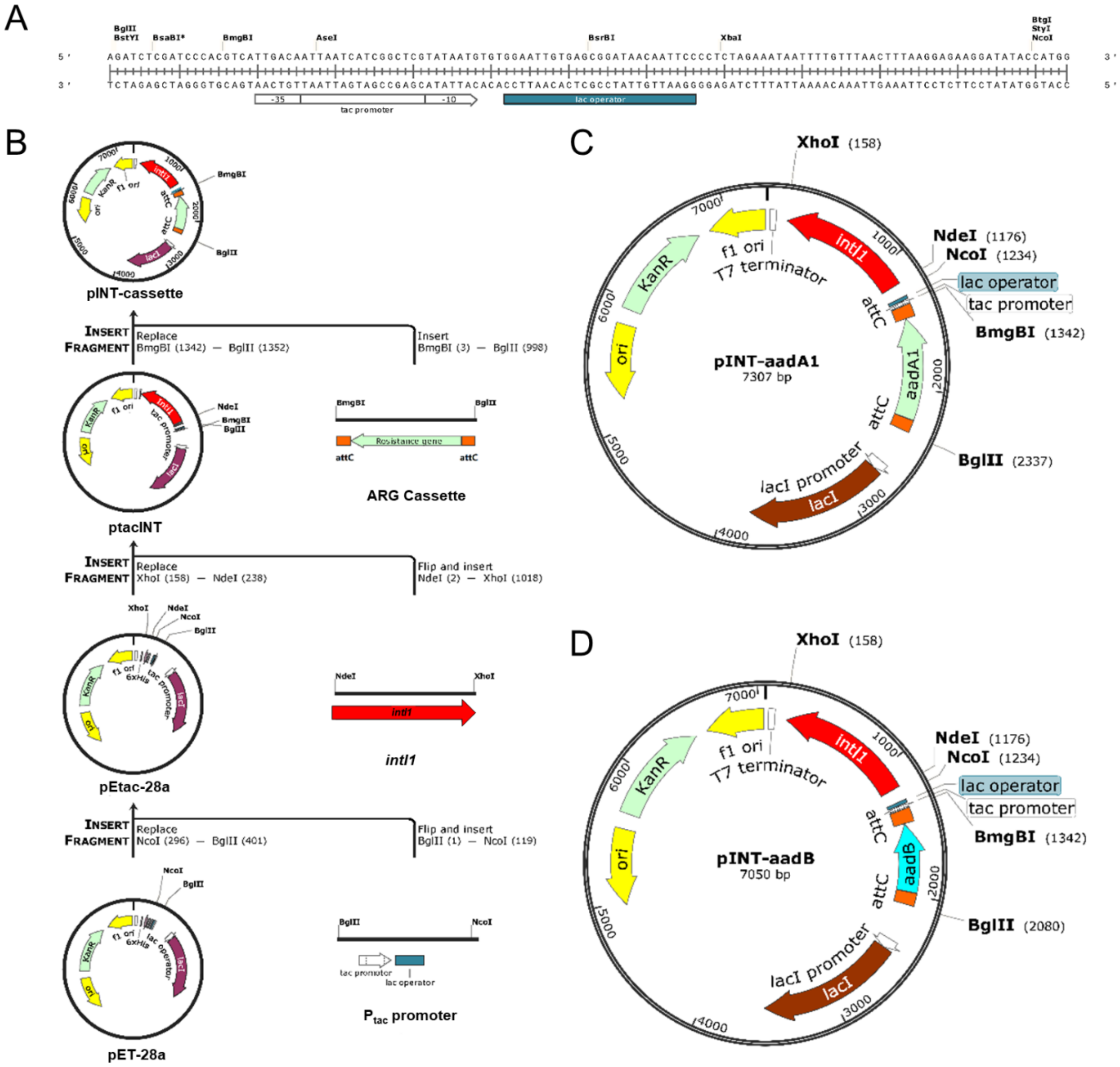

Fig S4 Schematic diagram of construction of pINT-cassette. (**A**) Sequence of BmgBI-P_{tac} for recombinant plasmid pINT-cassette. (**B**) Schematic diagram of pINT-cassette. (**C**-**D**) The resulting recombinant plasmids pINT-*aadA1* (C) and pINT-*aadB* (D).

Table S1 Strains and vectors used in this study.

Vectors/Strains	Description	Source
**Vectors**		
**R388**	33 kb conjugative plasmid containing a class 1 integron with *dfrB2-orfA* cassette array, TMP^{R}, SUL^{R}	(1) Stokes& Hall,1989
**plv-dCas9-sgRNA**	CRISPRi encoding vector, CM^{R}	(2) Lv et al. 2015
**plv-dCas9-R0**	CRISPRi recombinant plasmid, sgRNA R0, CM^{R}	This study
**plv-dCas9-R1**	CRISPRi recombinant plasmid, sgRNA R1, CM^{R}	This study
**plv-dCas9-R2**	CRISPRi recombinant plasmid, sgRNA R2, CM^{R}	This study
**plv-dCas9-R3**	CRISPRi recombinant plasmid, sgRNA R3, CM^{R}	This study
**plv-dCas9-R4**	CRISPRi recombinant plasmid, sgRNA R4, CM^{R}	This study
**plv-dCas9-R5**	CRISPRi recombinant plasmid, sgRNA R5, CM^{R}	This study
**plv-dCas9-R6**	CRISPRi recombinant plasmid, sgRNA R6, CM^{R}	This study
**pET-28a**	Cloning vector, KAN^{R}	Novagen
**pEtac-28a**	pET-28a with original T7 promoter replaced by *tac* promoter, KAN^{R}	This study
**ptacINT**	pEtac-28a with *intI1* gene overexpressing under *tac* promoter, KAN^{R}	This study
**pINT-*aadA1***	ptacINT with *aadA1* cassette added, KAN^{R}	This study
**pINT-*aadB***	ptacINT with *aadB* cassette added, KAN^{R}	This study
**R388-*aadA1***	R388 with *aadA1* cassette inserted, TMP^{R}, SUL^{R}, STR^{R}	This study
**R388-*aadB***	R388 with *aadB* cassette inserted, TMP^{R}, SUL^{R}, GEN^{R}	This study
**Strains**		

***E. coli* C600**	Wild-type strain, F^- *ton*A21 *thi*$^-$1 *thr*$^-$1 *leu*B6 *lac*Y1 *gln*V44 *rfb*C1 *fhu*A1 λ^-	Lab stock
***E. coli* Top10**	Competent cells	Biomed
***E. coli* DH5α(R388)**	*E. coli* DH5α carrying plasmid R388	Lab stock
***E. coli* ATCC 25922**	Quality control strain for determination of antibiotic resistance	ATCC
***Salmonella sp.*S084**	Wild-type strain of *aadB* cassette	Lab stock
***Salmonella sp.*S010**	Wild-type strain of *aadA1* cassette	Lab stock
***E. coli* C600(R388+plv-dCas9-R0)**	Recombinant *E. coli* C600 carrying R388 and plv-dCas9-R0	This study
***E. coli* C600(R388+plv-dCas9-R1)**	Recombinant *E. coli* C600 carrying R388 and plv-dCas9-R1	This study
***E. coli* C600(R388+plv-dCas9-R2)**	Recombinant *E. coli* C600 carrying R388 and plv-dCas9-R2	This study
***E. coli* C600(R388+plv-dCas9-R3)**	Recombinant *E. coli* C600 carrying R388 and plv-dCas9-R3	This study
***E. coli* C600(R388+plv-dCas9-R4)**	Recombinant *E. coli* C600 carrying R388 and plv-dCas9-R4	This study
***E. coli* C600(R388+plv-dCas9-R5)**	Recombinant *E. coli* C600 carrying R388 and plv-dCas9-R5	This study
***E. coli* C600(R388+plv-dCas9-R6)**	Recombinant *E. coli* C600 carrying R388 and plv-dCas9-R6	This study
***E. coli* C600(R388+plv-dCas9-R0+pINT-*aadA1*)**	Donor *E. coli* C600 carrying R388, plv-dCas9-R0 and pINT-*aadA1*	This study
***E. coli* C600(R388+plv-dCas9-R1+pINT-*aadA1*)**	Donor *E. coli* C600 carrying R388, plv-dCas9-R1 and pINT-*aadA1*	This study
***E. coli* C600(R388+plv-dCas9-R2+pINT-*aadA1*)**	Donor *E. coli* C600 carrying R388, plv-dCas9-R2 and pINT-*aadA1*	This study

**_E. coli_ C600(R388+plv-dCas9-R3+pINT-_aadA1_)**	Donor _E. coli_ C600 carrying R388, plv-dCas9-R3 and pINT-_aadA1_	This study
**_E. coli_ C600(R388+plv-dCas9-R4+pINT-_aadA1_)**	Donor _E. coli_ C600 carrying R388, plv-dCas9-R4 and pINT-_aadA1_	This study
**_E. coli_ C600(R388+plv-dCas9-R5+pINT-_aadA1_)**	Donor _E. coli_ C600 carrying R388, plv-dCas9-R5 and pINT-_aadA1_	This study
**_E. coli_ C600(R388+plv-dCas9-R6+pINT-_aadA1_)**	Donor _E. coli_ C600 carrying R388, plv-dCas9-R6 and pINT-_aadA1_	This study
**_E. coli_ C600(R388+plv-dCas9-R0+pINT-_aadB_)**	Donor _E. coli_ C600 carrying R388, plv-dCas9-R0 and pINT-_aadB_	This study
**_E. coli_ C600(R388+plv-dCas9-R1+pINT-_aadB_)**	Donor _E. coli_ C600 carrying R388, plv-dCas9-R1 and pINT-_aadB_	This study
**_E. coli_ C600(R388+plv-dCas9-R2+pINT-_aadB_)**	Donor _E. coli_ C600 carrying R388, plv-dCas9-R2 and pINT-_aadB_	This study
**_E. coli_ C600(R388+plv-dCas9-R3+pINT-_aadB_)**	Donor _E. coli_ C600 carrying R388, plv-dCas9-R3 and pINT-_aadB_	This study
**_E. coli_ C600(R388+plv-dCas9-R4+pINT-_aadB_)**	Donor _E. coli_ C600 carrying R388, plv-dCas9-R4 and pINT-_aadB_	This study
**_E. coli_ C600(R388+plv-dCas9-R5+pINT-_aadB_)**	Donor _E. coli_ C600 carrying R388, plv-dCas9-R5 and pINT-_aadB_	This study
**_E. coli_ C600(R388+plv-dCas9-R6+pINT-_aadB_)**	Donor _E. coli_ C600 carrying R388, plv-dCas9-R6 and pINT-_aadB_	This study
**_E.coli_ J53**	Wild-type recipient	Lab stock
**_E. coli_ J53 (R388)**	Transconjugant _E. coli_ J53 carrying R388	This study
**_E. coli_ J53 (R388-_aadA1_)**	Transconjugant _E. coli_ J53 carrying R388-_aadA1_	This study
**_E. coli_ J53 (R388-_aadB_)**	Transconjugant _E. coli_ J53 carrying R388-_aadB_	This study

TMP, trimethoprim; SUL, sulfamethoxazole; CM, chloramphenicol; KAN, kanamycin; STR, streptomycin; GEN, gentamicin; ATCC, American type culture collection.

Table S2 Oligonucleotides and primers used in this study.

Primers	Sequences (5'-3')	Vectors
**Oligonucleotides used for sgRNA synthesis**		
BspQI-R0-F	aaaTTCGACGTGAACTTGGTACG	plv-dCas9-R0
R0-BspQI-R	aacCGTACCAAGTTCACGTCGAA	
BspQI-R1-F	aaaCCAGTTGACATAAGCCTGTT	plv-dCas9-R1
R1-BspQI-R	aacAACAGGCTTATGTCAACTGG	
BspQI-R2-F	aaaCACACCGTGGAAACGGATGA	plv-dCas9-R2
R2-BspQI-R	aacTCATCCGTTTCCACGGTGTG	
BspQI-R3-F	aaaAGAACCTTGACCGAACGCAG	plv-dCas9-R3
R3-BspQI-R	aacCTGCGTTCGGTCAAGGTTCT	
BspQI-R4-F	aaaGCTGCGTTCGGTCAAGGTTC	plv-dCas9-R4
R4-BspQI-R	aacGAACCTTGACCGAACGCAGC	
BspQI-R5-F	aaaCGTGCCTTCATCCGTTTCCA	plv-dCas9-R5
R5-BspQI-R	aacTGGAAACGGATGAAGGCACG	
BspQI-R6-F	aaaCCGAACAGGCTTATGTCAAC	plv-dCas9-R6
R6-BspQI-R	aacGTTGACATAAGCCTGTTCGG	
**Primers used for construction of pINT-cassette**		
NdeI-intI1-F	attactaacaacatatgAAAACCGCCACTGCGCCGTTACC	ptacINT
intI1-XhoI-R	tatactcgagCTACCTCTCACTAGTGAGGGGCGGC	
BglII-aadA1-F	aatagatctGGTTAACAAGTGGCAGC	pINT-*aadA1*
aadA1-BmgBI-R	acaagatctGACCTGATAGTTTGGCTG	
BglII-aadB-F	cgtagatctAAGCAGCAAGCGCGTTA	pINT-*aadB*
aadB-BmgBI-R	ataagatctGGCCGAGCGCTGGAATA	
**Primers used for colony PCR**		
BspQI-R0-F	aaaTTCGACGTGAACTTGGTACG	plv-dCas9-R0

sgRNA-CX-R	GCGGAATATATCCCTAGGCCTGCAG	
BspQI-R1-F	aaaCCAGTTGACATAAGCCTGTT	plv-dCas9-R1
sgRNA-CX-R	GCGGAATATATCCCTAGGCCTGCAG	
BspQI-R2-F	aaaCACACCGTGGAAACGGATGA	plv-dCas9-R2
sgRNA-CX-R	GCGGAATATATCCCTAGGCCTGCAG	
BspQI-R3-F	aaaAGAACCTTGACCGAACGCAG	plv-dCas9-R3
sgRNA-CX-R	GCGGAATATATCCCTAGGCCTGCAG	
BspQI-R4-F	aaaGCTGCGTTCGGTCAAGGTTC	plv-dCas9-R4
sgRNA-CX-R	GCGGAATATATCCCTAGGCCTGCAG	
BspQI-R5-F	aaaCGTGCCTTCATCCGTTTCCA	plv-dCas9-R5
sgRNA-CX-R	GCGGAATATATCCCTAGGCCTGCAG	
BspQI-R6-F	aaaCCGAACAGGCTTATGTCAAC	plv-dCas9-R6
sgRNA-CX-R	GCGGAATATATCCCTAGGCCTGCAG	
intI1-F	TGATGGCGACGCACGACA	R388
intI1-R	TCCTGGCTGGCGAACGAG	
BglII-aadA1-F	aatagatctGGTTAACAAGTGGCAGC	pINT-*aadA1*
intI1-CX-R	ATCCGGATATAGTTCCTCCTTTCAGCA AAAAACC	
BglII-aadB-F	cgtagatctAAGCAGCAAGCGCGTTA	pINT-*aadB*
intI1-CX-R	ATCCGGATATAGTTCCTCCTTTCAGCA AAAAACC	
**Primers used for DNA sequencing**		
sgRNA-CX-F	TGTTTGTCGGTGAACGCTCTCTACTAG	plv-dCas9-R(0-6)
sgRNA-CX-R	GCGGAATATATCCCTAGGCCTGCAG	
intI1-CX-F	GAAGGATATACCATGGGCAGCAGCC	ptacINT
intI1-CX-R	ATCCGGATATAGTTCCTCCTTTCAGCA AAAAACC	
Cassette-CX-F	GGCTGCTGCCCATGGTATATCCTTC	pINT-cassette

Cassette-CX-R	CACCATACCCACGCCGAAACAAGCG	
Integration-CX-F	ACGAACCCAGTTGACATAAG	
aadA1-CX-R	TCGATGACGCCAACTAC	R388-*aadA1*
Integration-CX-F	ACGAACCCAGTTGACATAAG	
aadB-CX-R	GGCAGATTTCGCTCATCTG	R388-*aadB*
**Primers used for RT-qPCR**		
16S rRNA-RT-F	CCTACGGGAGGCAGCAG	-
16S rRNA-RT-R	ATTACCGCGGCTGCTGG	
dCas9-RT-F	TGGTGGAAGAAGACAAGAA	plv-dCas9-R(0-6)
dCas9-RT-R	CGCAGATGATAGATAGTTGGA	
intI1-RT-F	ATCGTTTGTTCGCCCAGCTT	pINT-cassette and R388
intI1-RT-R	GTGCCGTGATCGAAATCCAG	
drfB2-RT-F	AGAAATCTGGTGCCGCTTG	R388
drfB2-RT-R	ACTGAGCCTGGGTGGGATT	
sul1-RT-F	ACGGTGTTCGGCATTCTGA	R388
sul1-RT-R	TCCGACTCGCAGCATTTCA	

Primers were designed by Primer Premier 5.0 software using sequences downloaded from NCBI (http://www.ncbi.nlm.nih.gov/) as templates. The nucleotides labeled with gray background are the restriction endonuclease sites.

Table S3 IC_{50} of TMP and SUL in recombinant *E. coli* with/without aTc induction.

Strains	aTc	IC_{50} of TMP (μg/ml)	IC_{50} of SUL (μg/ml)
E. coli ATCC25922	-	2	32
E. coli C600(R388)	-	512	4096
E. coli C600(R388+plv-dCas9-R0)	-	512	4096
	+	512	4096
E. coli C600(R388+plv-dCas9-R1)	-	512	4096
	+	64	1024
E. coli C600(R388+plv-dCas9-R2)	-	512	4096
	+	64	256
E. coli C600(R388+plv-dCas9-R3)	-	512	4096
	+	64	128
E. coli C600(R388+plv-dCas9-R4)	-	512	4096
	+	64	2048
E. coli C600(R388+plv-dCas9-R5)	-	512	4096
	+	64	2048
E. coli C600(R388+plv-dCas9-R6)	-	512	4096
	+	64	128

IC_{50}, half-maximal inhibitory concentration; TMP, trimethoprim; SUL, sulfamethoxazole; aTc, anhydrotetracycline.

1. Stokes HW, Hall RM. 1989. A novel family of potentially mobile DNA elements encoding site-specific gene-integration functions: integrons. Mol Microbiol 3:1699-1683.
2. Lv L, Ren YL, Chen JC, Wu Q, Chen GQ. 2015. Application of CRISPRi for prokaryotic metabolic engineering involving multiple genes, a case study: controllable P(3HB-co-4HB) biosynthesis. Metab Eng 29:160-8.
